# Associations Between Agency and Sexual and Reproductive Health Communication in Early Adolescence: A Cross-cultural, Cross-sectional Study

**DOI:** 10.1016/j.jadohealth.2020.02.026

**Published:** 2020-09

**Authors:** Leah R. Koenig, Mengmeng Li, Linnea A. Zimmerman, Patrick Kayembe, Chaohua Lou, Eric Mafuta, José Ortiz, Caroline Moreau

**Affiliations:** a Department of Population, Family, and Reproductive Health, Johns Hopkins Bloomberg School of Public Health, Baltimore, Maryland; b Department of Epidemiology and Biostatistics, School of Public Health, Kinshasa School of Public Health, University of Kinshasa, Kinshasa, the Democratic Republic of the Congo; c National Health Commission Key Lab of Reproduction Regulation (Shanghai Institute of Planned Parenthood Research), Fudan University, Shanghai, China; d Faculty of Medical Sciences, University of Cuenca, Cuenca, Ecuador

**Keywords:** Sexual and reproductive health, Communication, Early adolescence, Empowerment, Agency

## Abstract

**Purpose:**

To assess the extent to which adolescents aged 10–14 have communicated about sexual relationships, pregnancy, and contraception and how agency in the form of voice and decision-making along with an enabling socioecological environment are associated with sexual and reproductive health (SRH) communication.

**Methods:**

Using data from the Global Early Adolescent Study, we included 1,367, 697, and 1,424 adolescents in Kinshasa, Cuenca, and Shanghai, respectively. Patterns of SRH communication and agency levels were described by site and sex. Multivariable logistic regressions assessed odds of SRH communication first in relation to socioecological characteristics and second with levels of agency, after adjustment for social environmental factors. Interaction terms tested sex differences in associations.

**Results:**

Experiences of SRH communication ranged from one in ten in Kinshasa to about half in Cuenca. Pregnancy was the most discussed SRH topic. Socioecological factors consistently related to SRH communication included older age and pubertal onset, while others varied by context. In multivariable analyses, voice was linked to all forms of SRH communication in Kinshasa and Cuenca with adjusted odds ratios ranging from 1.6 to 2.2, but not in Shanghai. In Cuenca, decision-making was associated with a 50% and 60% increase in odds of communication about pregnancy and contraception, respectively. In Kinshasa, a stronger association between voice and pregnancy discussions was observed for girls than boys.

**Conclusions:**

Developmental characteristics and voice were linked to communication about SRH among young adolescents across two contexts. Results suggest agency may play a role in shaping antecedents, like communication, to sexual behaviors.

**Implications and Contribution**

This study provides novel evidence about the links between empowerment and sexual and reproductive health (SRH) skills in early adolescence by documenting experiences of SRH communication and their associations with agency and developmental factors.

In recent years, investment in the sexual and reproductive health (SRH) [1] of adolescents and the empowerment of women and girls [2] have emerged as critical steps on the global health and development agendas. Each has been highlighted as a means toward addressing immediate health needs and promoting SRH across the life course. While adolescent SRH programs have largely focused on improving adolescents' knowledge and enhancing the availability and quality of services provided to them, there is a growing focus on the ability of girls to make informed decisions about their reproductive health based on their environments [3].

SRH communication is a skill essential to young adolescents' accumulation of knowledge and arrival at partnered sex prepared, aware of risk and both HIV and pregnancy prevention, and able to negotiate their needs, boundaries, and desires. Existing research has linked young people's formal (within school or healthcare settings) and interpersonal (with parents or caregivers, friends, and sexual partners) SRH communication to positive behaviors and outcomes, including service utilization [4], condom and contraceptive awareness [5], and use [6].

One aspect that has not been extensively explored is the degree to which SRH communication relies on young adolescents' empowerment. A body of work has examined relationships between empowerment levels and SRH outcomes among adult women and adolescent girls aged 15 and older. However, few of these studies conceptualize empowerment as a multidimensional construct that lies at the interplay between attributes and external resources that interacts to inform the extent to which individuals are able to achieve their SRH goals. Instead, the vast majority have relied on proxy indicators of agency, ranging from household decision-making to political participation to sexual self-efficacy [7]. A recent review of the literature on family planning and women's empowerment found that only 3% of identified articles employed a multidimensional measure to assess empowerment, which may better reflect the complex nature of the construct than any single indicator or dimension [8].

To our knowledge, extant literature examining associations of empowerment-related factors with SRH among adolescents is scarce. Existing analyses on this topic primarily employ measures of perceived behavioral control, reflecting adolescents' anticipated self-efficacy to achieve SRH outcomes like communication [9,10]. For example, Schouten et al. operationalized perceived behavioral control as adolescents' ease of and opportunity to discuss SRH, demonstrating associations between perceived behavioral control and frequency of SRH communication with parents [11]. Meanwhile, Thoma et al. found links between perceived behavioral control (defined as confidence in, lack of difficulty with, and autonomy in decision-making about condom use) and discussions about condoms with mothers among young men who have sex with men [12]. While this existing research has begun to explore the connections between SRH communication and SRH self-efficacy outcomes among adolescents, it does not consider how agency expressed in other dimensions of daily life may inform SRH. Furthermore, this research largely overlooks the early adolescent period, a critical window for SRH skill-building before the age of first sex for many young people. In addition, while recognition of the role that boys and men play in shaping women's and girls' agency is rising [13], empowerment research continues to focus nearly exclusively on girls and women. Understanding these linkages between agency and reproductive health among both young adolescent boys and girls will help to expand understanding about gendered divisions of power and their relationships to SRH, and about these important links among adolescent boys themselves.

One of the factors limiting studies of how young people are empowered to voice their needs and to make decisions surrounding SRH is the lack of a cross-cultural indicator assessing agency at this developmental stage. To address this gap and empirically assess the contribution of agency in healthy transitions from early to older adolescence, the Global Early Adolescent Study (GEAS) has developed a multidimensional measure of agency including dimensions of mobility, voice, and decision-making power that are salient to young people's lives across diverse cultural settings. The psychometric properties of the instrument that have been tested among nearly 2,000 adolescents in 15 cities are presented elsewhere [14]. Building on this research, we seek to understand how these dimensions of agency in early adolescence relate to SRH communication.

Recognizing that the exercise of agency is shaped by relational, community, and other environmental factors, indicators are often understood in conjunction with the socioecological environment [15]. Indeed, the GEAS scales of agency are considered within the context of opportunity structures to construct actualized empowerment. While the social factors contributing to adolescents' SRH communication [16,17] and outcomes [18]—such as age, puberty, family structure, parental relationships, and perceptions of the community—have been explored extensively in the literature, this study examines how a combination of these social factors interact with dimensions of agency to inform SRH outcomes.

To address these gaps in knowledge, this analysis employs data from the GEAS, a global study on norms about gender and their relationship to health and well-being beginning in early adolescence. Using baseline data collected in three sites, the objectives of the present study are to (1) describe patterns of SRH communication across sites and by sex, (2) examine associations between adolescents' socioecological environments and SRH communication, and (3) assess whether agency in the form of either voice or decision-making power relates to young people's history of communication about SRH, adjusting for the social environment, and whether this association is consistent by sex and across contexts.

## Methodology

### Study context

This comparative study employs data from three urban low-resource settings in Kinshasa, Democratic Republic of the Congo (DRC); Cuenca, Ecuador; and Shanghai, China. The contexts of these study sites vary considerably. In Kinshasa, a city of over 11 million where adolescents comprise a quarter of the population, young people often grow up in challenging environments [19,20]. Nearly half of Kinshasa's population subsists on less than a dollar a day [21], and 7% of adolescents have dropped out of school at lower secondary school [22]. The city of Cuenca has approximately 600,000 inhabitants; about 2% of the population meets the criteria for income poverty [23], and 40% of its population are under age 20 [23]. Five percent of adolescents in the southern region of Ecuador, where Cuenca lies, are estimated to be out of school at lower secondary school [22]. In contrast, Shanghai is a megacity of over 24 million, with a small share of its population receiving governmental financial support [24]. Twelve percent of Shanghai's population is under the age of 17, and rates of school dropout are under 1% [25].

### Sampling

Adolescents aged 10–14 were surveyed in disadvantaged urban areas of three cities (Kinshasa, DRC; Cuenca, Ecuador; and Shanghai, China), as part of the GEAS between June 2017 and March 2018. In Kinshasa, both in-school and out-of-school adolescents were included in the original study due to interest among stakeholders in studying these issues among out-of-school adolescents. Probability and multistaged sampling used to select in-school and out-of-school participants, respectively. Participants in Cuenca were recruited using probability sampling from schools, stratified by age and sex. In Shanghai, all eligible students in grades 6–8 were recruited from three purposively selected public schools.

After obtaining parental consent and adolescent assent, adolescents completed the ninety-minute survey using tablets, by face-to-face interview (Kinshasa, due to low literacy) or computer-assisted self-interview (Cuenca and Shanghai). Research protocols were approved by each site's institutional ethical review committee and approved or deemed exempt for secondary data analysis by the Johns Hopkins School of Public Health institutional review board.

The initial samples included 2,842 adolescents in Kinshasa, 704 in Cuenca, and 1,760 in Shanghai. In Kinshasa, we considered only adolescents in the control arm of a broader quasi-experimental study for inclusion (n = 1,381 cases), to keep consistency with the other sites and to avoid any introduction of bias due to self-selection into the intervention. We then excluded individuals missing all three SRH communication outcomes (n = 6 in Kinshasa, n = 2 in Cuenca, n = 152 in Shanghai) or missing more than 30% of items used to construct the agency subscales (n = 8 in Kinshasa, n = 5 in Cuenca, n = 184 in Shanghai). Applying these exclusion criteria, 1.0% of cases were dropped in Kinshasa, 1.0% in Cuenca, and 19.1% in Shanghai. Given the large share of cases excluded in Shanghai, we assessed and noted differences between the included and excluded subsamples by age, sex distribution, educational attainment, caregiver monitoring, and perceived neighborhood safety (Appendix Table 1). After exclusion, our analytical samples were comprised of 1,367 adolescents in Kinshasa, 697 in Cuenca, and 1,424 in Shanghai.

### Measures

Two of the three GEAS cross-cultural domains of agency were considered for this analysis: voice (seven items measuring the extent to which young people can express their opinions and be heard) and decision-making (four items measuring adolescents' ability to make choices autonomously in their daily lives). The items, response options, and internal reliability for each scale are presented in Appendix Table 2. Each of the scales ranged from 1 to 4, with a higher score indicating greater agency. Due to skewed distributions of agency mean scores and an effort to keep consistency in analytical strategies across sites, we dichotomized the continuous mean scores at their medians within each site to identify adolescents with “high” or “low” voice and decision-making power in logistic regressions.

Covariates were self-reported at various levels of the ecological environment. Individual sociodemographic factors included adolescents' age, binary indicators of sex, pubertal onset (prepubertal vs. pubertal), and educational attainment (behind in school or out of school vs. at or above expected grade level for age). Family characteristics included binary variables for parental structure (living with both parents vs. with one parent or other relatives) as well as caregiver closeness, monitoring, and migration. Peer and neighborhood-level covariates included a binary variable indicating time typically spent with close friends (no close friends or no time spent with friends weekly vs. saw friends once a week or more), as well as binary indicators reflecting social cohesion (based on an aggregate measure of four items measuring trust and solidarity among neighbors), and whether or not participants feel safe in their neighborhoods.

Three individual items were used to assess whether adolescents had ever discussed three sexual and reproductive health topics with anyone: sexual relationships, pregnancy and how it occurs, or contraception.

### Data analysis

We first conducted exploratory analysis to evaluate patterns of missingness across all items comprising the two agency subscales and excluded observations that met the exclusion criteria (outlined in the Methods section). For the remaining samples, we used k-nearest neighbor (kNN) imputation to impute missing agency responses (with k-values of 31, 25, and 37 in Kinshasa, Cuenca, and Shanghai, respectively) followed by imputation to account for missing data on covariates (kNN with k-values of 36, 22, and 31 in Kinshasa, Cuenca, and Shanghai, respectively) [26].

SRH communication patterns were examined overall by site and by sex, while agency and ecological factors were described by site, sex, and SRH communication outcomes using chi-squared, Fisher exact, and Student *t*-tests. We examined bivariate associations between ecological factors (individual, family, peer, and neighborhood levels) and the three SRH communication outcomes as well as those between agency (voice and decision-making) and SRH communication. Multivariable logistic regressions assessed the independent effect of each ecological factor on SRH communication and subsequently evaluated the effect of agency levels on SRH communication, adjusting for all ecological covariates. Collinearity among covariates was assessed by the variance inflation factor value and no multicollinearity was noted. All analyses were stratified by site to assess similarities and differences in these associations by context. Interactions between agency and sex were also tested in the latter multivariable models in order to assess sex differences in the relationships between agency and SRH communication. kNN imputation was conducted using RStudio (RStudio, Inc., Boston, MA); all other analyses were conducted using Stata/SE 15.1 (StataCorp LLC, College Station, TX).

## Results

Table 1 summarizes sample characteristics. On average, adolescents were older in Shanghai than in the other two sites. Parental structure differed between contexts; just over half of adolescents in Kinshasa and over three quarters of those in Shanghai were living with both parents at the time of the survey. Caregiver migration was less common in Cuenca (22.1% compared to about half in the other two sites). Neighborhood perceptions were most positive in Shanghai (56.9% reported high social cohesion and 96.8% felt safe in their neighborhood).

**Figure 1:**
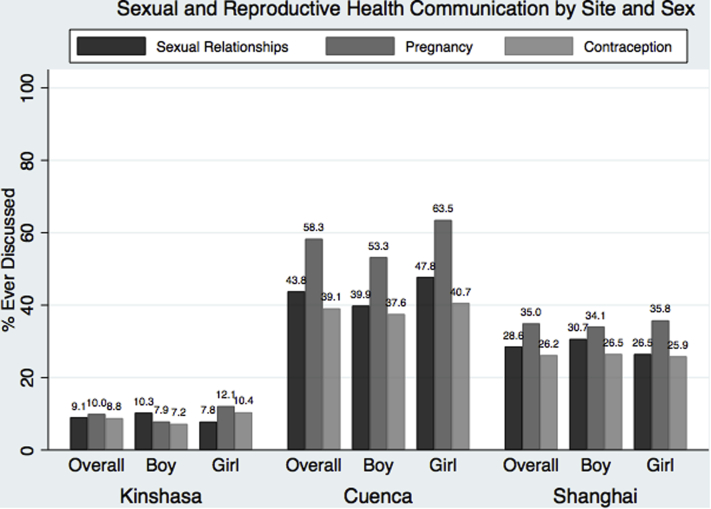
Sexual and reproductive health communication by site and sex.

SRH communication patterns varied by site and sex (Figure 1). Adolescents in Cuenca were the most likely to have talked to anyone about sexual relationships (43.8%), pregnancy (58.3%), or contraception (39.1%), while only 1 in 10 had ever discussed each topic in Kinshasa. In Kinshasa, girls were more likely than boys to have discussed both pregnancy (12.1% vs. 7.9%, *p* = .009) and contraception (10.4% vs. 7.2%, *p* = .042). In Cuenca, more girls than boys had discussed sexual relationships (47.8% vs. 39.9%, *p* = .036) and pregnancy (63.5% vs. 53.3%, *p* = .006). No differences by sex were detected in Shanghai.

**Figure 2:**
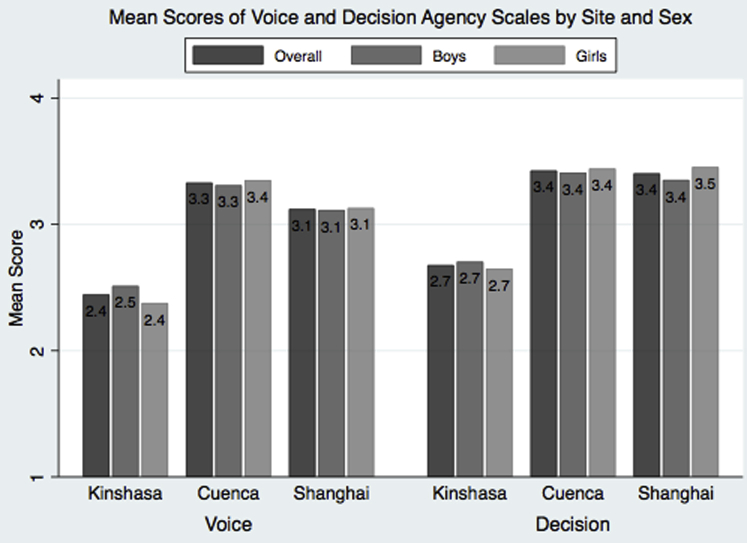
Mean scores of voice and decision agency scales by site and sex.

Levels of agency also differed by sex and site (Figure 2). Mean scores for voice and decision-making were lowest in Kinshasa (2.4 for voice and 2.7 for decision-making). Scores for voice were higher for boys than girls in Kinshasa (2.5 vs. 2.4, *p* < .001) and comparable by sex in the other two sites. Decision-making mean scores were higher for girls than boys in Shanghai (3.5 vs. 3.4, *p* = .003), while no sex differences were observed in Kinshasa and Cuenca.

Distributions of ecological factors by the three SRH communication outcomes are outlined in Appendix Table 3. We next examined the results of bivariate logistic regression analysis for the individual, family, peer, and community factors predictive of SRH communication. In each of the three sites, older age and pubertal onset were associated with increased odds of communication about all three SRH topics. These results were confirmed in multivariable analysis, with the odds of SRH communication increasing between 17% and 74% across topics and sites with older age and ranging between 2.2- and 3.7-fold across topics in Cuenca and Kinshasa among adolescents with pubertal onset (Table 2).

**Table 1:** Sample description

	Kinshasa (N = 1,367)	Cuenca (N = 697)	Shanghai (N = 1,424)
	Mean ± standard deviation		
Individual			
Age	12.0 ± 1.4	11.9 ± 1.4	12.5 ± 1.0
	col %		
Girl	50.2%	49.9%	50.4%
At age expected school grade or higher	57.9%	99.7%	83.4%
Pubertal onset	51.5%	77.8%	88.3%
Family			
Living with both parents	56.9%	66.4%	83.7%
Close to caregiver	63.1%	76.9%	56.4%
High caregiver monitoring	40.7%	75.6%	84.1%
Caregiver migrated	41.9%	22.5%	55.1%
Peer			
See friends at least once a week	93.9%	60.8%	59.7%
Neighborhood			
High neighborhood cohesion	26.0%	40.7%	56.9%
Feels safe in neighborhood	79.4%	82.2%	96.8%

**Table 2:** Crude and adjusted odds ratios of sexual and reproductive health communication, by site

Kinshasa	Sexual relationships				Pregnancy				Contraception			
	OR	95% CI	aOR	95% CI	OR	95% CI	aOR	95% CI	OR	95% CI	aOR	95% CI
Age	1.9***	**1.6–2.2**	1.6***	**1.4–1.9**	1.8***	**1.6–2.1**	1.5***	**1.3–1.8**	1.7***	**1.5–2.0**	1.6***	**1.3–1.9**
Sex												
Boy	Ref		Ref		Ref		Ref		Ref		Ref	
Girl	.7	.5–1.1	.5**	**.4–.8**	1.6**	**1.1–2.3**	1.1	.7–1.7	1.5*	**1.0–2.2**	1.2	.7–1.8
Education												
Behind in or out of school	Ref		Ref		Ref		Ref		Ref		Ref	
Age expected grade or higher	.9	.6–1.3	1.2	.8–1.8	1.1	.8–1.6	1.5	1.0–2.2	1.8**	**1.2–2.8**	2.2**	**1.4–3.4**
Puberty												
Prepubertal	Ref		Ref		Ref		Ref		Ref		Ref	
Pubertal	3.9***	**2.5–6.1**	3.2***	**1.9–5.4**	6.3***	**3.9–10.3**	3.7***	**2.1–6.4**	4.1***	**2.5–6.5**	2.2**	**1.3–3.9**
Parental structure												
No parents or one parent only	Ref		Ref		Ref		Ref		Ref		Ref	
Both parents	.8	.5–1.1	.8	.6–1.2	.7*	**.5–.9**	.6*	**.4–.9**	1.0	.7–1.4	.9	.6–1.4
Closeness with caregiver												
No/no caregiver	Ref		Ref		Ref		Ref		Ref		Ref	
Yes	.8	.6–1.2	1.2	.8–1.7	.5***	**.4–.7**	.7*	**.5–1.0**	.8	.5–1.2	.9	.6–1.4
Neighborhood safety												
Does not feel safe in neighborhood	Ref		Ref		Ref		Ref		Ref		Ref	
Feel safe in neighborhood	.7*	**.4–1.0**	.6*	**.4–.9**	1.0	.6–1.5	.9	.6–1.4	1.0	.6–1.6	.8	.5–1.4

Cuenca	Sexual relationships				Pregnancy				Contraception			
	OR	95% CI	aOR	95% CI	OR	95% CI	aOR	95% CI	OR	95% CI	aOR	95% CI
Age	1.5***	**1.4–1.7**	1.5***	**1.3–1.7**	1.6***	**1.4–1.8**	1.5***	**1.3–1.7**	1.8***	**1.6–2.1**	1.7***	**1.5–2.0**
Sex												
Boy	Ref		Ref		Ref		Ref		Ref		Ref	
Girl	1.4*	**1.0–1.9**	1.2	.9–1.7	1.5**	**1.1–2.1**	1.3	.9–1.9	1.1	.8–1.6	1.1	.8–1.6
Puberty												
Prepubertal	Ref		Ref		Ref		Ref		Ref		Ref	
Pubertal	4.1***	**2.7–6.3**	2.5***	**1.6–4.1**	4.4***	**3.0–6.4**	2.7***	**1.7–4.2**	3.7***	**2.4–5.8**	2.2**	**1.3–3.6**
Caregiver migration												
No/no caregiver	Ref		Ref		Ref		Ref		Ref		Ref	
Caregiver migrated	.6*	**.4–.9**	.6**	**.4–.9**	.7*	**.5–1.0**	.7	.5–1.0	.7**	**.5–1.0**	.6*	**.4–1.0**
Closeness with caregiver												
No/no caregiver	Ref		Ref		Ref		Ref		Ref		Ref	
Yes	1.3	.9–1.8	1.7*	**1.1–2.5**	1.0	.7–1.5	1.3	.9–2.0	.9	.6–1.3	1.2	.8–1.8
Caregiver monitoring												
Low/no caregiver	Ref		Ref		Ref		Ref		Ref		Ref	
High	1.7**	**1.2–2.5**	1.4	.9–2.1	1.5*	**1.1–2.1**	1.2	.8–1.7	1.0	.7–1.5	.8	.5–1.2

Shanghai	Sexual relationships				Pregnancy				Contraception			
	OR	95% CI	aOR	95% CI	OR	95% CI	aOR	95% CI	OR	95% CI	aOR	95% CI
Age	1.2**	**1.1–1.4**	1.2*	**1.0–1.3**	1.3***	**1.1–1.5**	1.3***	**1.1–1.4**	1.3***	**1.1–1.5**	1.2**	**1.1–**1**.4**
Education												
Behind in or out of school	Ref		Ref		Ref		Ref		Ref		Ref	
Age expected grade or higher	1.2	.9–1.7	1.2	.9–1.7	1.4*	**1.0**–**1.9**	1.3*	**1.0**–**1.8**	1.2	.9–1.7	1.2	.8–1.7
Puberty												
Prepubertal	Ref		Ref		Ref		Ref		Ref		Ref	
Pubertal	1.5*	**1.0**–**2.3**	1.4	.9–2.1	1.5*	**1.0**–**2.1**	1.1	.8–1.7	1.6*	**1.0**–**2.4**	1.3	.9–2.1
Closeness with caregiver												
No/no caregiver	Ref		Ref		Ref		Ref		Ref		Ref	
Yes	.5***	**.4**–**.7**	.6***	**.5**–**.8**	.7**	**.6**–**.9**	.8*	**.6**–**1.0**	.6***	**.5**–**.8**	.7**	**.5**–**.9**
Caregiver monitoring												
Low/no caregiver	Ref		Ref		Ref		Ref		Ref		Ref	
High	.7*	**.5**–**.9**	.9	.6–1.2	1.0	.7–1.3	1.1	.8–1.6	.8	.6–1.1	.9	.7–1.3
Time spent per week with close friends												
No close friends or no time	Ref		Ref		Ref		Ref		Ref		Ref	
See friends at least once a week	1.3*	**1.0**–**1.7**	1.3*	**1.0**–**1.7**	1.3**	**1.1**–**1.7**	1.3*	**1.0**–**1.7**	1.5**	**1.1**–**1.9**	1.4**	**1.1**–**1.9**
Neighborhood cohesion												
Low neighborhood cohesion	Ref		Ref		Ref		Ref		Ref		Ref	
High neighborhood cohesion	.7**	**.5**–**.9**	.7**	**.6**–**.9**	.8*	**.6**–**1.0**	.8	.6–1.0	.8	.6–1.0	.8	.7–1.1
Neighborhood safety												
Does not feel safe in neighborhood	Ref		Ref		Ref		Ref		Ref		Ref	
Feel safe in neighborhood	.5*	**.3**–**.9**	.6	.3–1.1	.4**	**.2**–**.8**	.5*	**.3**–**.9**	.3***	**.2**–**.6**	.4**	**.2**–**.7**

Models for Kinshasa also adjusted for caregiver migration, caregiver monitoring, time spent with close friends per week, and neighborhood cohesion, none of which were significantly related to SRH communication.

Models for Cuenca also adjusted for education attainment, parental structure, time spent with close friends per week, neighborhood cohesion, and neighborhood safety, none of which were significantly related to SRH communication.

Models for Shanghai also adjusted for sex, parental structure, and caregiver migration, none of which were significantly related to SRH communication.

Numbers in bold indicates statistical significance.

OR, odds ratio; CI, confidence interval; aOR, adjusted odds ratio; Ref, reference.

*p < .05; **p < .01; ***p < .001.

Additional factors related to SRH communication were heterogeneous across sites in bivariate and multivariable analyses (Table 2). In Kinshasa, adolescents with higher education performance were more likely to have communicated about pregnancy and about contraception before and after adjustment. Adolescents living with both parents and those who reported feeling close to their parents were less likely to report communication about pregnancy. Perceived neighborhood insecurity was linked to lower likelihood of having discussed sexual relationships. Meanwhile, adolescents whose parents were born in Cuenca were more likely to discuss all SRH topics. In Shanghai, low parental closeness was related to communication about the three SRH topics, while time spent with friends was linked to higher odds of having discussed all three topics. Those in Shanghai who reported low cohesion among their community were more likely to have discussed sexual relationships, and participants who felt threatened in their neighborhood were also more likely to report they had discussed SRH.

**Figure 3:**
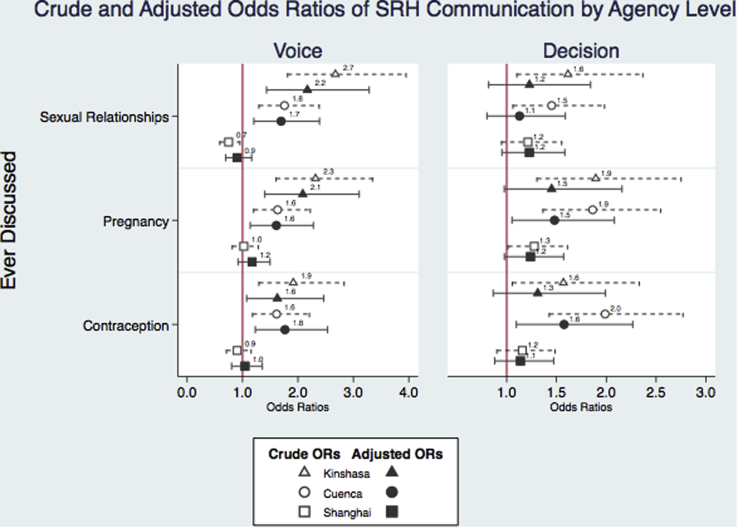
Crude and adjusted odds ratios of SRH communication by agency level.

Bivariate logistic regressions assessing odds of SRH communication by levels of voice and decision-making revealed links between both voice and decision-making and SRH communication in Kinshasa and Cuenca (Figure 3 and Appendix Table 4). After adjustment for socioecological factors at the individual, family, peer, and neighborhood levels, more voice was related to higher odds of communication about all three SRH topics in both Kinshasa and Cuenca with adjusted odds ratios ranging from 1.6 to 2.2. No significant relationship between voice and SRH communication was observed in Shanghai after adjustment. Significance tests of interaction terms revealed possible differential effects of voice on communication about pregnancy by sex in Kinshasa (*p* = .017), with a significantly stronger association among girls than boys (adjusted relative odds ratio [aOR] for girls vs. boys, 2.1; 95% confidence interval [CI], 1.2–3.7). Fewer associations were observed between decision-making capacity and SRH communication. Adolescents with high decision-making scores were more likely to have talked about pregnancy (aOR, 1.5; 95% CI, 1.0–2.1) and contraception (aOR, 1.6; 95% CI, 1.1–2.3) in Cuenca. No significant differences in the associations of SRH communication and decision-making were detected by sex.

## Discussion

This analysis found SRH communication among young adolescents was relatively uncommon, associated with developmental factors across sites, and linked in two contexts to adolescents' ability to voice their needs and opinions. Decision-making was related to history of pregnancy and contraception discussions among adolescents in Cuenca. Particularly low levels of SRH communication were observed in Kinshasa, within the context of high fertility [27] and a low modern contraceptive prevalence rate [20] among adolescents in the DRC. Prior research has demonstrated substantial social barriers to these conversations in household and school settings in the DRC [28]. Across sites, pregnancy was the most commonly discussed SRH topic, followed by sexual relationships and contraception. These patterns echo evidence from prior investigations into parent-child SRH communication, which found that these conversations tend to focus on pregnancy risk and abstinence, and less frequently address contraception [29].

In Kinshasa and Cuenca, more girls than boys had discussed most SRH topics, a finding that reflects the social and biological consequences of sexual activity that disproportionately impact girls [30,31], which may prompt more SRH conversations with girls. Other studies have highlighted parents' prioritization of SRH discussions with their daughters as a result of a sexual double standard, views that boys' sexual activity is inevitable, and greater fear for their daughters' safety in sexual encounters [32,33]. Such sex differences in these discussions may be particularly pronounced in highly patriarchal societies.

Our analysis also examined the role of the socioecological environment in shaping SRH communication, in recognition its actualization can be promoted or constrained by one's context. Congruent with prior research, increased age [34] and puberty [35] were strongly associated with SRH communication. Older age increased participants' likelihood of having discussed each of the SRH topics across contexts, as these topics grow more relevant to adolescents' emerging sexual lives. Independently, our study found that puberty was the characteristic most strongly linked to SRH communication in Cuenca and Kinshasa. At the same time that young adolescents may be more curious about sex at the onset of puberty than they were in the prior period [17,35], they may also be engaged in discussions about sexual and reproductive health topics by their parents, who worry about the health and social consequences of sexual activity as they mature [36].

This study found additional patterns of individual, family, peer, and neighborhood factors linked with SRH communication that were heterogeneous across sites. Factors predominantly on the individual level in Kinshasa, family level in Cuenca, and family, peer, and neighborhood levels in Shanghai were influential upon SRH communication. These findings indicate the varied cultural, social, and structural factors that impact SRH communication in each setting and indicate differential implications for interventions to create an enabling environment.

These varying results across sites also underscore the varied contexts in which such discussions can take place, within family or school settings, or in the neighborhood with peers. Our findings additionally call attention to the agents who may transmit SRH information of varied quality to adolescents, information that ranges in accuracy and in its framing of sexuality and reproductive health. Additional research is needed to more extensively explore the nature of SRH communication interactions in early adolescence and how this communication contributes to SRH knowledge.

Beyond the role of ecological factors in shaping SRH communication, a key finding to emerge from our study is the relationship between SRH communication and both increased decision-making power in Cuenca and greater voice in Kinshasa and Cuenca, after adjustment for the socioecological environment. Our findings, if corroborated by additional research, suggest a role for agency-promoting interventions in setting positive SRH trajectories for both boys and girls in the adolescent period. While promoting empowerment is a long-term and complex process, in the short-term, interventions such as comprehensive sexuality education should pay special attention to adolescents who are less able to voice their opinions or influence decision-making to ensure they receive important SRH information even if they are less likely than others to initiate such a conversation. On the other hand, the associations found here may indicate that discussion of SRH may play a role in promoting adolescents' perception of their own power by building foundational negotiation skills and knowledge. Regardless of the direction of this relationship, our study builds upon a small body of prior findings that link SRH-specific agency indicators to SRH outcomes during the adolescent period [11,37].

This study found considerable variation in agency, experiences of SRH communication, and the relationships between the two across three very distinctive urban contexts. While we found links between all SRH communication and voice in Kinshasa and Cuenca, decision-making was only associated with communication about pregnancy and contraception in Cuenca. In addition, no relationships between these factors were found in Shanghai. Differences in these findings across the three sites could be attributed to context-specific factors such as the influence of religiosity in Cuenca and Kinshasa, the existence of Machismo culture in Latin America, and the impact of the One-Child Policy on girls' empowerment in Shanghai [38,39]. These elements shape gender roles, expressions of empowerment among adolescent boys and girls, norms about SRH communication, and ultimately these findings. Within more conservative social contexts, adolescents may need greater agency to overcome social barriers to the conversations about SRH that young people in other settings can more easily navigate.

The present analysis is also novel in its inclusion of boys and comparison of the examined relationships by sex. While the field has focused on girls' preparedness for sexual activity, the present results challenge the assumption that boys arrive at sexual activity both more empowered and more prepared than girls, as levels of SRH communication were largely comparable by sex in these three settings. While we found that voice was more strongly associated with communication about pregnancy for girls than boys in Kinshasa, notably no other associations between SRH communication and agency differed significantly by sex. Our results also contribute novel evidence by employing a validated measure of overall agency among young adolescents to evaluate such associations, suggesting that a broader concept of agency may relate to SRH skills.

Several limitations of this study should be noted. First, findings are not generalizable beyond our study areas. Second, our measure of SRH communication only described whether or not adolescents reported having discussed each SRH topic. These measures lacked specificity about the people adolescents talked to, contexts in which they took place, content, temporality, frequency or quality of these discussions, which likely impact their relationship to agency, and to downstream SRH behaviors and outcomes [40]. Indicators were self-reported and therefore subject to social desirability bias. Third, the large share (19%) of the Shanghai sample excluded due to missing data and observed differences between the included and excluded cases may have introduced unaccounted bias into our analyses. Fourth, the cross-sectional associations observed cannot be interpreted as causational. Therefore, a longitudinal assessment of these relationships, which may be carried out using subsequent waves of the GEAS, is necessary to better understand the directionality of observed associations in their contribution to healthy behaviors as adolescents become sexually active.

Taken together, our findings suggest that empowerment factors, including the enabling environment and dimensions of agency, are linked to communication about SRH in the early adolescent period in certain contexts. We conclude that further research, with representative samples, longitudinal data and more specific SRH communication items that allow for disaggregation between types of SRH communication, is needed to fully understand the role of empowerment in shaping SRH trajectories during early adolescence.

## References

[1] Lane C., Brundage C., Kreinin T. (2017). Why We Must invest in early adolescence: Early intervention, Lasting impact. J Adolesc Health.

[2] Every Woman Every Child The global Strategy for women’s, Children’s and adolescents’ health (2016–2030): Survive, thrive, Transform. http://globalstrategy.everywomaneverychild.org/pdf/EWEC_globalstrategyreport_200915_FINAL_WEB.pdf.

[3] Edmeades J., Hinson L., Sebany M., Murithi L. (2018). A conceptual Framework for reproductive empowerment: Empowering individuals and Couples to Improve their health (Brief).

[4] Hall K.S., Moreau C., Trussell J. (2012). Associations between sexual and reproductive health communication and health service use among U.S. adolescent women. Perspect Sex Reprod Health.

[5] Melaku Y.A., Berhane Y., Kinsman J., Reda H.L. (2014). Sexual and reproductive health communication and awareness of contraceptive methods among secondary school female students, northern Ethiopia: A cross-sectional study. BMC public health.

[6] Widman L., Choukas-Bradley S., Noar S.M. (2016). Parent-adolescent sexual communication and adolescent safer sex behavior: A Meta-analysis. JAMA Pediatr.

[7] Malhotra A., Schuler S.R. (2005). Women’s empowerment as a variable in international development. *Meas Empower Cross-Discip*. Perspect.

[8] Prata N., Fraser A., Huchko M.J. (2017). Women’s empowerment and family planning: A review of the literature. J Biosoc Sci.

[9] Lefkowitz E.S., Boone T.L., Shearer C.L. (2004). Communication with best friends about sex-related topics during emerging adulthood. J Youth Adolesc.

[10] Wang B., Stanton B., Deveaux L. (2014). The impact of parent involvement in an effective adolescent risk reduction intervention on sexual risk communication and adolescent outcomes. AIDS Educ Prev Off Publ Int Soc AIDS Educ.

[11] Schouten B.C., van den Putte B., Pasmans M., Meeuwesen L. (2007). Parent–adolescent communication about sexuality: The role of adolescents’ beliefs, subjective norm and perceived behavioral control. Patient Educ Couns.

[12] Thoma B.C., Huebner D.M. (2018). Parent-adolescent communication about sex and condom Use among young men who have sex with men: An examination of the theory of planned behavior. Ann Behav Med Publ Soc Behav Med.

[13] Barker G., Ricardo C., Nascimento M. (2007). Engaging men and boys in Changing gender-based Inequity in Health: Evidence from Programme interventions. https://apps.who.int/iris/handle/10665/43679.

[14] Zimmerman L.A., Li M., Moreau C. (2019). Measuring agency as a dimension of empowerment among young adolescents globally; findings from the Global Early Adolescent Study. SSM Popul Health.

[15] Alsop R, Heinsohn N. Measuring empowerment in Practice: Structuring analysis and framing indicators. 10.1596/1813-9450-3510

[16] Dessie Y., Berhane Y., Worku A. (2015). Parent-adolescent sexual and reproductive health communication is very limited and associated with adolescent poor behavioral beliefs and subjective norms: Evidence from a community based cross-sectional study in Eastern Ethiopia. PLoS One.

[17] Muhwezi W.W., Katahoire A.R., Banura C. (2015). Perceptions and experiences of adolescents, parents and school administrators regarding adolescent-parent communication on sexual and reproductive health issues in urban and rural Uganda. Reprod Health.

[18] Kalolo A., Mazalale J., Krumeich A., Chenault M. (2019). Social cohesion, social trust, social participation and sexual behaviors of adolescents in rural Tanzania. BMC Public Health.

[19] World Bank (2017). Democratic Republic of Congo Urbanization review: Productive and inclusive cities for an emerging Democratic Republic of Congo.

[20] (2017). Performance monitoring and Accountability 2020 Kinshasa, DRC adolescents & young adults health Brief.

[21] Population distribution by province of the DRC, 2010 - Democratic Republic of the Congo data Portal. https://drcongo.opendataforafrica.org/ayyfgdd/population-distribution-by-province-of-the-drc-2010.

[22] World Inequality Database on Education. https://www.education-inequalities.org.

[23] National Institute of Statistics and Census INEC open data bank. http://aplicaciones3.ecuadorencifras.gob.ec/BIINEC-war/index.xhtml.

[24] Guo F., Cheng Z. (2010). Labour Market Disparity, poverty, and Inequality in urban China. China Perspect.

[25] Office of Shanghai Statistics Bureau 20.4 basic Statistics of various Phases education in Main Years. http://tjj.sh.gov.cn/tjnj/nje18.htm?d1=2018tjnje/E2004.htm.

[26] Beretta L., Santaniello A. (2016). Nearest neighbor imputation algorithms: A critical evaluation. BMC Med Inform Decis Mak.

[27] (2015). Performance monitoring and Accountability 2020 survey Round 3, PMA2015/DRC-R3 (Kinshasa) Snapshot of indicators.

[28] Mbadu M., Gahungu N., Wood F., Bertrand J. (2018). Attitudes toward sexual and reproductive health among adolescents and young people in urban and rural DR Congo. Reprod Health.

[29] Seif S.A., Kohi T.W., Moshiro C.S. (2017). Caretaker-adolescent communication on sexual and reproductive health: A cross-sectional study in Unguja-Tanzania Zanzibar. BMC Public Health.

[30] Mmari K., Moreau C., Gibbs S.E. (2018). ‘Yeah, I’ve grown; I can’t go out anymore’: Differences in perceived risks between girls and boys entering adolescence. Cult Health Sex.

[31] Ninsiima A.B., Leye E., Michielsen K. (2018). “Girls have more challenges; they need to Be Locked up”: A Qualitative study of gender norms and the sexuality of young adolescents in Uganda. Int J Environ Res Public Health.

[32] O’Sullivan L.F., Meyer-Balhburg H.F.L., Watkins B.X. (2000). Social cognitions associated with pubertal development in a sample of urban, low-income, African-American and Latina girls and mothers. J Adolesc Health.

[33] Wilson E.K., Koo H.P. (2010). Mothers, fathers, sons, and daughters: Gender differences in factors associated with parent-child communication about sexual topics. Reprod Health.

[34] Tesso D.W., Fantahun M.A., Enquselassie F. (2012). Parent-young people communication about sexual and reproductive health in E/Wollega zone, west Ethiopia: Implications for interventions. Reprod Health.

[35] Ritchwood T.D., Metzger I.W., Powell T.W. (2019). How does pubertal development impact caregiver-adolescent communication about sex in rural, African American Families? An Examination of Mediation effects. J Early Adolesc.

[36] Manu A.A., Mba C.J., Asare G.Q. (2015). Parent-child communication about sexual and reproductive health: Evidence from the Brong Ahafo region, Ghana. Reprod Health.

[37] Kalolo A., Kibusi S.M. (2015). The influence of perceived behaviour control, attitude and empowerment on reported condom use and intention to use condoms among adolescents in rural Tanzania. Reprod Health.

[38] De Meyer S., Jaruseviciene L., Zaborskis A. (2014). A cross-sectional study on attitudes toward gender equality, sexual behavior, positive sexual experiences, and communication about sex among sexually active and non-sexually active adolescents in Bolivia and Ecuador. Glob Health Action.

[39] Fong V.L. (2002). China’s one-child policy and the empowerment of urban daughters. Am Anthropologist.

[40] Rogers A.A., Ha T., Stormshak E.A., Dishion T.J. (2015). Quality of parent-adolescent conversations about sex and adolescent sexual behavior: An observational study. J Adolesc Health Off Publ Soc Adolesc Med.

